# Bavachin Induces Ferroptosis through the STAT3/P53/SLC7A11 Axis in Osteosarcoma Cells

**DOI:** 10.1155/2021/1783485

**Published:** 2021-10-18

**Authors:** Yi Luo, Xu Gao, Luetao Zou, Miao Lei, Junming Feng, Zhenming Hu

**Affiliations:** ^1^Department of Orthopedics, The First Affiliated Hospital of Chongqing Medical University, Chongqing 400016, China; ^2^Department of Pain Medicine, Bishan Hospital of Chongqing, Chongqing 402760, China; ^3^Department of Ophthalmology, Bishan Hospital of Chongqing, Chongqing 402760, China; ^4^Department of Pathology, Bishan Hospital of Chongqing, Chongqing 402760, China

## Abstract

Ferroptosis is a new form of regulated cell death, which is mediated by intracellular iron. Although it is reported that bavachin has antitumour effects on several tumour cells and prompts the reactive oxygen species (ROS) generation, it is unclear whether ferroptosis can be induced by bavachin in osteosarcoma (OS) cells. In this study, we found that bavachin inhibits the viability of MG63 and HOS OS cell lines along with an increase in the ferrous iron level, ROS accumulation, malondialdehyde overexpression, and glutathione depletion. Moreover, iron chelators (deferoxamine), antioxidants (Vit E), and ferroptosis inhibitors (ferrostatin-1 and liproxstatin-1) reverse bavachin-induced cell death. Bavachin also altered the mitochondrial morphology of OS cells, leading to smaller mitochondria, higher density of the mitochondrial membrane, and reduced mitochondrial cristae. Further investigation showed that bavachin upregulated the expression of transferrin receptor, divalent metal transporter-1, and P53, along with downregulating the expression of ferritin light chain, ferritin heavy chain, p-STAT3 (705), SLC7A11, and glutathione peroxidase-4 in OS cells. More importantly, STAT3 overexpression, SLC7A11 overexpression, and pretreatment with pifithrin-*α* (P53 inhibitor) rescued OS cell ferroptosis induced by bavachin. The results show that bavachin induces ferroptosis via the STAT3/P53/SLC7A11 axis in OS cells.

## 1. Introduction

Osteosarcoma (OS) is the most frequent bone tumour in children and adolescents, in addition to being the most common aggressive malignancy originating from mesenchymal cells [1, 2]. Although neoadjuvant chemotherapy combined with surgery and postoperative chemotherapy has been used to treat OS, the five-year survival rate of OS is not satisfactory on account of development of early metastases and chemotherapy resistance [3, 4]. Thus, it is imperative to find new targets and pharmaceuticals to improve the prognosis.

Ferroptosis is a new form of regulated cell death (RCD) that is mediated by intracellular iron and is quite different from other forms of cell death, such as apoptosis, autophagy, or necrosis [5]. Intracellular iron reacts with H_2_O_2_ through Fenton reaction (Fe^2+^ + H_2_O_2_→Fe^3+^ + HO·+ OH^−^) to generate many reactive oxygen species (ROS) and trigger lipid peroxidation (LP) to induce ferroptosis [6]. Aberrant iron metabolism, ROS generation, and abnormal LP are the hallmarks of ferroptosis [5, 7]. Morphologically, ferroptotic cells demonstrate shrunken mitochondria, higher density of the mitochondrial membrane, and reduced or diminished mitochondrial cristae [8]. Additionally, ferroptosis can be inhibited by iron chelators or antioxidants and activated by some small compounds (erastin), or by inhibiting glutathione peroxidase-4 (GPX4) [9]. The cystine/glutamate antiporter system Xc^−^, which is composed of two subunits, SLC7A11 (light chain) and SLC3A2 (heavy chain), is closely linked to ferroptosis [10]. SLC7A11, which transports cystine into the cells, enhances glutathione (GSH) synthesis, further promoting the inhibition of ferroptosis by GPX4. P53, as an upstream mediator of SLC7A11, mediates the repression of SLC7A11 to initiate ferroptosis in tumour cells [11]. In addition to breast cancer, colorectal cancer, and anaplastic thyroid cancer [12–14], ferroptosis also exerts anticancer effects in OS [15, 16]. Some clinical drugs including sulfasalazine and sorafenib have proven to induce ferroptosis to trigger anticancer effects [17], via inhibiting the system Xc^−^ [18, 19]. Therefore, activating P53 to downregulate SLC7A11/GPX4 to trigger ferroptosis could be a potential method to inhibit progression of OS cells.

Signal transducer and activator of transcription 3 (STAT3) belongs to the STAT family and is a vital transcription factor involved in inflammation and tumour progression [20]. Classical STAT3 activation involves the phosphorylation of STAT3 at Tyr705 (p-STAT3 (705)), which interacts with and inhibits P53 [21]. Wang et al. discovered that high expression of STAT3 is relevant to increased malignancy and poor prognosis [22]. As described above, it is critical to inactivate STAT3 to halt tumour progression.

Flavonoids have multiple biological functions, especially anticancer effects induced by the prooxidant activity [23]. Amentoflavone inhibits glioma cells via inducing ROS accumulation and ferroptosis [24]. Robustaflavone also induces ferroptosis by increasing ROS and lipid peroxidation in MCF-7 cells [25]. Bavachin belongs to flavonoids and is a bioactive compound extracted from the fruit of *Psoralea corylifolia* and displays various functions including anti-inflammatory, lipid-lowering, and cholesterol-reducing effects [26]. A previous study showed that bavachin stimulates osteoblast differentiation by activating the Wnt pathway and is considered an estrogen supplement [27]. Recently, it has been shown that bavachin also exerts antitumour effects. Bavachin has been demonstrated to inhibit human hepatocellular carcinoma cells by inducing apoptosis, accompanied by ROS accumulation [28]. It has been reported that bavachin inhibits melanoma cells by downregulating the MAPK signaling pathway [29]. Notably, bavachin was shown to trigger the apoptosis of multiple myeloma cells by inhibiting p-STAT3 (705) and increasing P53 [30]. Although it has been reported that bavachin can exacerbate ROS accumulation and suppress several tumour cells, it is unclear whether ferroptosis could be induced by bavachin, and if so, what is the underlying mechanism?

We hypothesized that bavachin increases intracellular ferrous iron, ROS, and malondialdehyde (MDA) levels and induces ferroptosis by the downregulation of SLC7A11 through inactivating STAT3 to upregulate P53. We hope that our research will provide promising pharmaceutical targets for the treatment of OS.

## 2. Materials and Methods

### 2.1. Reagents

Bavachin was purchased from MCE (China), and pifithrin-*α* (PFT-*α*), deferoxamine (DFO), ferrostatin-1 (Fer-1), and liproxstatin-1 (Lip-1) were purchased from Topscience (China). Vitamin E was purchased from Beyotime (Shanghai, China). Rabbit polyclonal anti-transferrin receptor antibody (TFRC, AF5343, 1 : 1000), rabbit polyclonal anti-divalent metal transporter-1 antibody (DMT1, DF12740. 1 : 1000), rabbit polyclonal anti-ferritin light chain antibody (FTL, DF6604, 1 : 1000), rabbit polyclonal anti-ferritin heavy chain antibody (FTH, DF6278, 1 : 1000), rabbit polyclonal anti-SLC7A11 antibody (DF12509, 1 : 1000), rabbit polyclonal anti-P53 antibody (AF0879, 1 : 1000), rabbit polyclonal anti-STAT3 antibody (AF6294, 1 : 1000), rabbit polyclonal anti-p-STAT3 (705) antibody (AF3293, 1 : 1000), and rabbit polyclonal anti-glutathione peroxidase-4 antibody (GPX4, DF6701, 1 : 1000) were purchased from Affinity Bioscience (China). HRP goat anti-rabbit IgG was purchased from Earthox (USA).

### 2.2. Cell Culture

The human OS cell lines MG63 and HOS were purchased from Procell (China). The cells were cultured in a humidified CO_2_ incubator at 37°C and 5% CO_2_ and grown in DMEM/high-glucose (Gibco, USA) supplemented with 10% foetal bovine serum (MRC, China).

### 2.3. Cell Viability

Cell viability was assessed using the Cell Counting Kit-8 (CCK-8) (APExBIO, China). According to the protocol, the absorbance values of the samples were read at 450 nm using a fluorescence microplate reader (Varioskan&LUX, Thermo Fisher, China). Subsequently, the cell death ratio was calculated using the formula: cell death ratio (%) = (*A*_sample_ − *A*_blank_)/(*A*_control_ − *A*_blank_) × 100.

### 2.4. Transmission Electron Microscope (TEM)

MG63 and HOS cells were collected and fixed in 2.5% glutaraldehyde in 0.1 M phosphate-buffered saline (PBS, pH 7.4) at room temperature for 24 h. Then, the cells were fixed in 2% osmium tetroxide in PBS at room temperature for 1 h. Next, the samples were dehydrated with a graded series of ethanol solutions (50% to 100%). Subsequently, the samples were embedded in epoxy resin and sectioned into ultrathin sections (60 nm). Finally, the ultrathin sections were steeped with uranyl acetate (1%) and lead citrate (0.1%) and observed and photographed using an H-7500 TEM (Hitachi, Japan).

### 2.5. Mitochondrial Membrane Potential (MMP) Detection

MMP was observed using JC-1 assay kit (Beyotime, China). According to the protocol, the cells were stained with the JC-1 probe and observed under a fluorescence microscope (DMIL4000, Leica, China). Green fluorescence indicates a low MMP, whereas red fluorescence indicates a high MMP.

### 2.6. Ferrous Iron Assay

Cellular ferrous iron levels were detected using FerroOrange (Dojindo, China). According to the protocol, the cells were incubated with FerroOrange for 0.5 h. Fluorescence intensity (Ex: 543 nm, Em: 580 nm) was assessed using a fluorescence microplate reader (Varioskan&LUX, Thermo Fisher, China). The ferrous iron levels were finally expressed as a ratio to the fluorescence intensity value of the control.

### 2.7. ROS Assay

Cellular ROS levels were measured using a commercial ROS assay kit (Beyotime, China). According to the protocol, the cells were loaded with a 2,7-dichlorodihydrofluorescein diacetate probe (10 *μ*M). The cells were then incubated at 37°C for 20 min. Finally, the fluorescence intensity (Ex: 488 nm, Em: 525 nm) was assessed using a fluorescence microplate reader (Varioskan&LUX, Thermo Fisher, China).

### 2.8. GSH Assay

GSH levels were assayed using the GSH assay kit (Nanjing Jiancheng, Nanjing, China). The harvested cells were crushed by sonication to obtain the supernatant used for measuring the GSH levels in a fluorescence microplate reader (Varioskan&LUX, Thermo Fisher, China). The levels of GSH were expressed as a ratio to the absorbance value of the control cells assessed at 405 nm.

### 2.9. Malondialdehyde (MDA) Assay

MDA levels were assayed using the MDA Assay Kit (Beyotime, China). First, the cells were lysed using the RIPA Lysis Buffer (Servicebio, China) with a protease inhibitor and centrifugated at 100,00 g for 10 min at 4°C to obtain the supernatant. The protein concentration of the samples was measured using the BCA Protein Assay Kit (Beyotime, China). Subsequently, the collected supernatant (0.1 ml) was added to thiobarbituric acid (0.2 ml) and incubated at 100°C for 15 min. Finally, the samples were read at 532 nm using a fluorescence microplate reader (Varioskan&LUX, Thermo Fisher, China). The MDA levels were expressed as a ratio to the absorbance value of the control.

### 2.10. Western Blotting Analysis

The cells were lysed and homogenized using RIPA Lysis Buffer (Servicebio, China) with a protease inhibitor. The protein concentration of the samples was measured using the BCA Protein Assay Kit (Beyotime, China). The proteins were added to the loading buffer (Beyotime, China) and denatured in boiling water for 5 min. Each protein sample (40 *μ*g) was loaded onto an SDS-PAGE (10%–12%) gel and transferred to PVDF membranes. The membranes were blocked with NcmBlot Blocking Buffer (NCM Biotech, China) and incubated with the primary antibodies at 4°C overnight. The membranes were subsequently incubated with the secondary antibody at room temperature for 1.5 h. Finally, the bands were developed using a HyperSignal ECL kit (4A Biotech, China).

### 2.11. Cell Transfection

For evaluating the overexpression of STAT3 and SLC7A11, an overexpressing plasmid along with a negative control (NC) plasmid was purchased from Vigene Biosciences (China). Briefly, cells were transfected with the plasmids using Lipofectamine 2000 (Invitrogen, China). After 48 h, the cells were subjected to subsequent experiments.

### 2.12. Statistical Analysis

Data are presented as means ± SD (*n* = 3). One-way ANOVA and Tukey's multiple comparisons test were used to analyze the data. All statistical analyses were performed using GraphPad Prism 7 (https://www.graphpad.com/). Statistical significance was set at *p* < 0.05.

## 3. Results

### 3.1. Bavachin Inhibits OS Cell Viability

**Figure 1:**
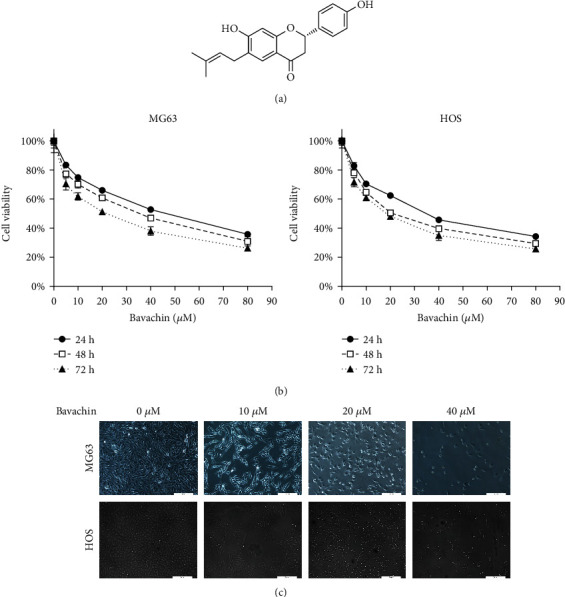
Bavachin inhibits osteosarcoma (OS) cell viability. (a) Chemical structure of bavachin. (b) CCK8 assay shows bavachin inhibits OS cells in a concentration- (5 *μ*M, 10 *μ*M, 20 *μ*M, 40 *μ*M, and 80 *μ*M) and time- (24 h, 48 h, and 72 h) dependent manner. (c) Morphologic features of OS cells on microscopy. Cells became round and showed shrunk after bavachin (10 *μ*M, 20 *μ*M, and 40 *μ*M) treatment for 24 h.

To evaluate the effects of bavachin on OS cells, MG63 and HOS cells were treated with bavachin (5 *μ*M, 10 *μ*M, 20 *μ*M, 40 *μ*M, and 80 *μ*M) for 24 h, 48 h, and 72 h, and then cell viability was measured using the CCK8 assay. As shown in Figure 1(b), bavachin inhibited the viability of both MG63 and HOS cells in a concentration- and time-dependent manner. According to CCK8 assay, the half-maximal inhibitory concentration (IC_50_) of bavachin at 24 h was determined. The IC_50_ values for MG63 and HOS cells were 42.32 *μ*M and 34.2 *μ*M, respectively. Therefore, 40 *μ*M of bavachin concentration was selected for the following experiments. Morphological changes in MG63 and HOS cells following bavachin treatment were observed. Light microscopy images showed that the shape of the cells became round, and there was an increase in cell shrinkage and cell death with increasing concentration of bavachin (Figure 1(c)).

### 3.2. Bavachin Induces Ferrous Iron Accumulation to Initiate OS Cell Death

**Figure 2:**
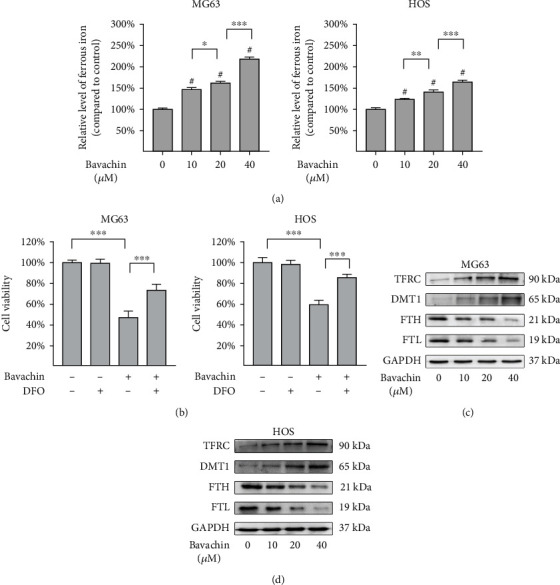
Bavachin-induced ferrous iron accumulation initiates death of osteosarcoma (OS) cells. (a) Bavachin (24 h) induced ferrous iron accumulation in OS cells occurs in a concentration-dependent manner. (b) Deferoxamine (DFO) rescues cells from bavachin-induced cell death. (c, d) Western blotting analysis shows the protein levels of transferrin receptor (TFRC), divalent metal transporter-1 (DMT1), ferritin light chain (FTH), and ferritin heavy chain (FTL) in MG63 and HOS cells treated with bavachin (10 *μ*M, 20 *μ*M, and 40 *μ*M) for 24 h. ^∗^*p* < 0.05, ^∗∗^*p* < 0.01, ^∗∗∗^*p* < 0.001, and ^#^*p* < 0.001 vs. the control group.

**Figure 3:**
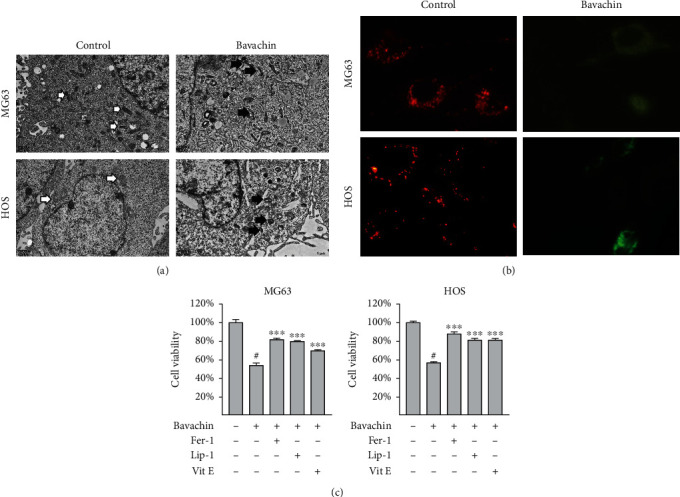
Bavachin induces ferroptosis in osteosarcoma (OS) cells. (a) The ultrastructure of MG63 and HOS cells was observed using transmission electronic microscopy. Cells in the bavachin (40 *μ*M, 24 h) group display shrinking mitochondria, higher density of the mitochondrial membrane, and reduction or disappearance of the mitochondrial cristae. (b) Merged images of JC-1 staining demonstrate the change in the mitochondrial membrane potential (MMP) (red: aggregates and high MMP; green: monomers and low MMP). (c) CCK8 assay shows that deferoxamine (DFO), ferrostatin-1 (Fer-1), and liproxstatin-1 (Lip-1) rescue bavachin-induced cell death in OS cells. ^∗∗∗^*p* < 0.001 vs. the bavachin group. ^#^*p* < 0.001 vs. the control group.

To evaluate whether bavachin affects ferrous iron accumulation, we assessed the expression of ferrous iron in OS cells treated with bavachin (10 *μ*M, 20 *μ*M, and 40 *μ*M) for 24 h. Compared to the control group, bavachin increased the ferrous iron concentration in a concentration-dependent manner (Figure 2(a)). As illustrated in Supplementary Figures1(a) and 1(b), ferrous iron levels reached maximum at 24 h of 40 *μ*M bavachin stimulation. Furthermore, OS cells pretreated with 100 *μ*M of DFO (iron chelator) for 1 h recovered from bavachin-induced (40 *μ*M, 24 h) cell death (Figure 2(b)). Finally, western blotting was used to explore the mechanism of ferrous iron accumulation induced by bavachin. As shown in Figures 3(c) and (d), bavachin treatment increased TFRC and DMT1 expressions and decreased FTH and FTL expression in a concentration-dependent manner (10 *μ*M, 20 *μ*M, and 40 *μ*M).

### 3.3. Bavachin Induces Ferroptosis in OS Cells

To demonstrate bavachin-induced ferroptosis in OS cells, we looked into the morphology of the treated cells. TEM showed that MG63 and HOS cells treated with 40 *μ*M bavachin for 24 h demonstrated shrinking mitochondria, higher density of the mitochondrial membrane, and reduction or disappearance of mitochondrial cristae, which are typical morphological features of ferroptosis (Figure 3(a)). Moreover, in fluorescence images of JC-1 staining, green fluorescence was observed in the bavachin (40 *μ*M for 24 h) group and red fluorescence in the control group (Figure 3(b)). Bavachin treatment reduced the MMP of OS cells compared with that of the control group. Finally, to clarify whether bavachin induced ferroptosis, OS cells were pretreated with ferroptosis inhibitors Fer-1 (5 *μ*M), Lip-1 (2 *μ*M), and Vit E (150 *μ*M) for 1 h. As shown in Figure 3(c), the results of the CCK8 assay showed that Fer-1, Lip-1, and Vit E rescued bavachin-induced OS cell death.

### 3.4. Bavachin Induces GSH Depletion and LP Accumulation in OS Cells

**Figure 4:**
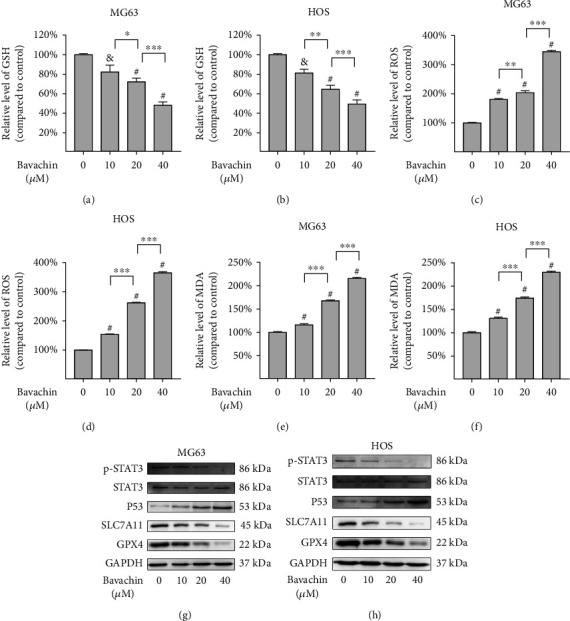
Bavachin induces GSH depletion and lipid peroxidation accumulation in osteosarcoma (OS) cells. (a, b) Bavachin induces GSH depletion in a dose-related manner over 24 h. (c, d) Bavachin greatly induces ROS generation in OS cells over 24 h. (e, f) Bavachin increases MDA accumulation in a dose-related manner. (g, h) Western blotting analysis shows the expression of p-STAT3, STAT3, P53, SLC7A11, and GPX4 in OS cells treated with bavachin (10 *μ*M, 20 *μ*M, and 40 *μ*M) for 24 h. ^&^*p* < 0.01 vs. the control group, ^#^*p* < 0.001 vs. the control group, ^∗^*p* < 0.05, ^∗∗^*p* < 0.01, and ^∗∗∗^*p* < 0.001.

The levels of GSH, ROS, and MDA were measured to determine whether bavachin induces oxidative stress in OS cells. It was found that the level of GSH declined gradually as bavachin concentration (10 *μ*M, 20 *μ*M, and 40 *μ*M) increased over 24 h (Figures 4(a) and 4(b)). However, ROS levels were remarkably upregulated by bavachin stimulation (Figures 4(c) and 4(d)). Moreover, ROS levels reached their highest values at 24 h of 40 *μ*M bavachin stimulation (Supplementary Figure 1(c) and 1(d)). As illustrated in Figures 4(e) and 4(f), bavachin markedly elevates the MDA level, which is a marker of LP, in a concentration-dependent manner for 24 h. To further analyze the molecular mechanism of bavachin-induced LP in OS cells, western blotting was performed to detect the expression of related proteins. The results demonstrated that bavachin inhibited GPX4, SLC7A11, and p-STAT3 expressions and increases P53 expressions in OS cells (Figures 4(g) and 4(h)).

### 3.5. SLC7A11 Overexpression Alleviates Bavachin-Induced Ferroptosis in OS Cells

**Figure 5:**
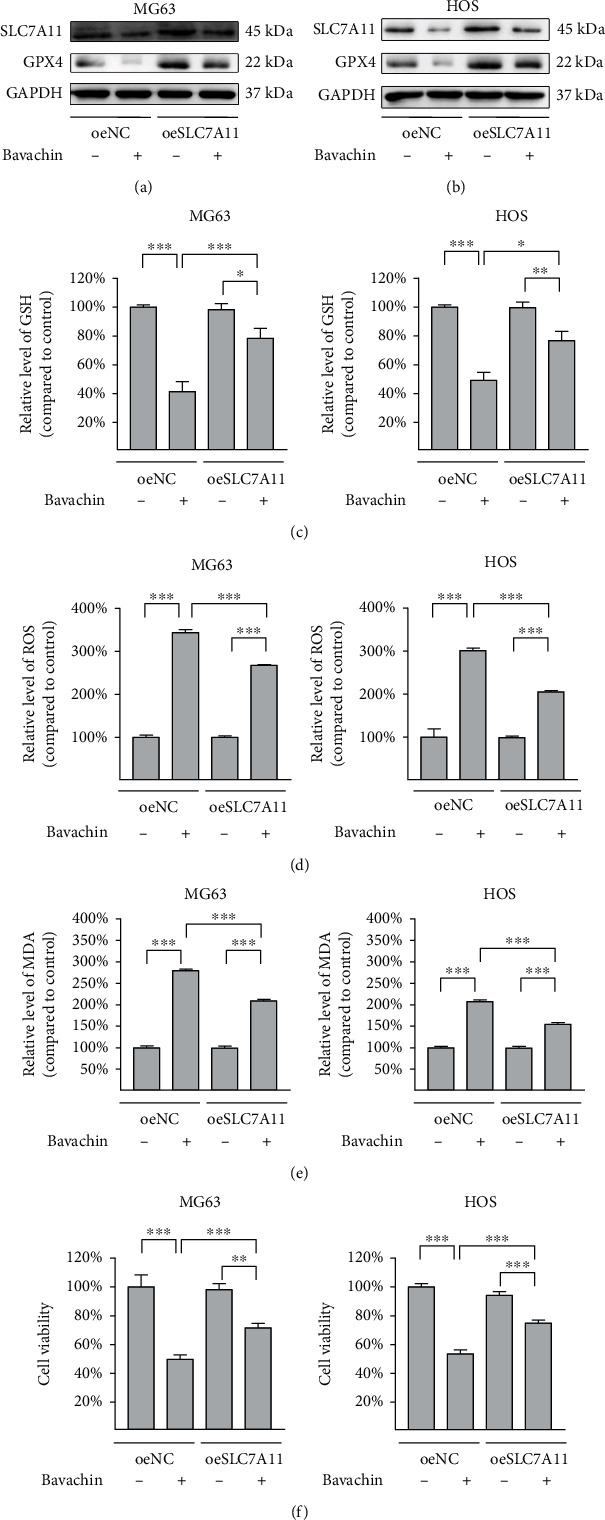
SLC7A11 overexpression alleviates bavachin-induced ferroptosis in osteosarcoma (OS) cells. OS cells were transfected with a SLC7A11 overexpressing plasmid and treated with bavachin (40 *μ*M, 24 h). (a, b) Western blotting demonstrates the relative levels of SLC7A11 and GPX4 protein expression. (c) Overexpression of SLC7A11 restores bavachin-induced GSH depletion in OS cells. (d, e) Overexpression of SLC7A11 reduces bavachin-induced ROS and MDA accumulation. (f) Overexpression of SLC7A11 recovers bavachin-induced ferroptosis in OS cells. ^∗^*p* < 0.05, ^∗∗^*p* < 0.01, and ^∗∗∗^*p* < 0.001.

OS cells were transfected with the SLC7A1 overexpressing plasmid to examine whether SLC7A1 overexpression could rescue bavachin-induced ferroptosis. As indicated in Figures 5(a)–5(c), SLC7A11 upregulation increases the decline in GPX4 expression and GSH content induced by bavachin (40 *μ*M, 24 h). SLC7A11 overexpression attenuates the bavachin-induced accumulation of ROS and MDA (Figures 5(d) and 5(e)). Importantly, SLC7A11 overexpression rescues bavachin-induced OS cell death (Figure 5(f)). Overall, SLC7A1 overexpression inhibits bavachin-induced ferroptosis in OS cells.

### 3.6. P53 Inactivation Upregulates SLC7A11 and Alleviates Bavachin-Induced Ferroptosis in OS Cells

**Figure 6:**
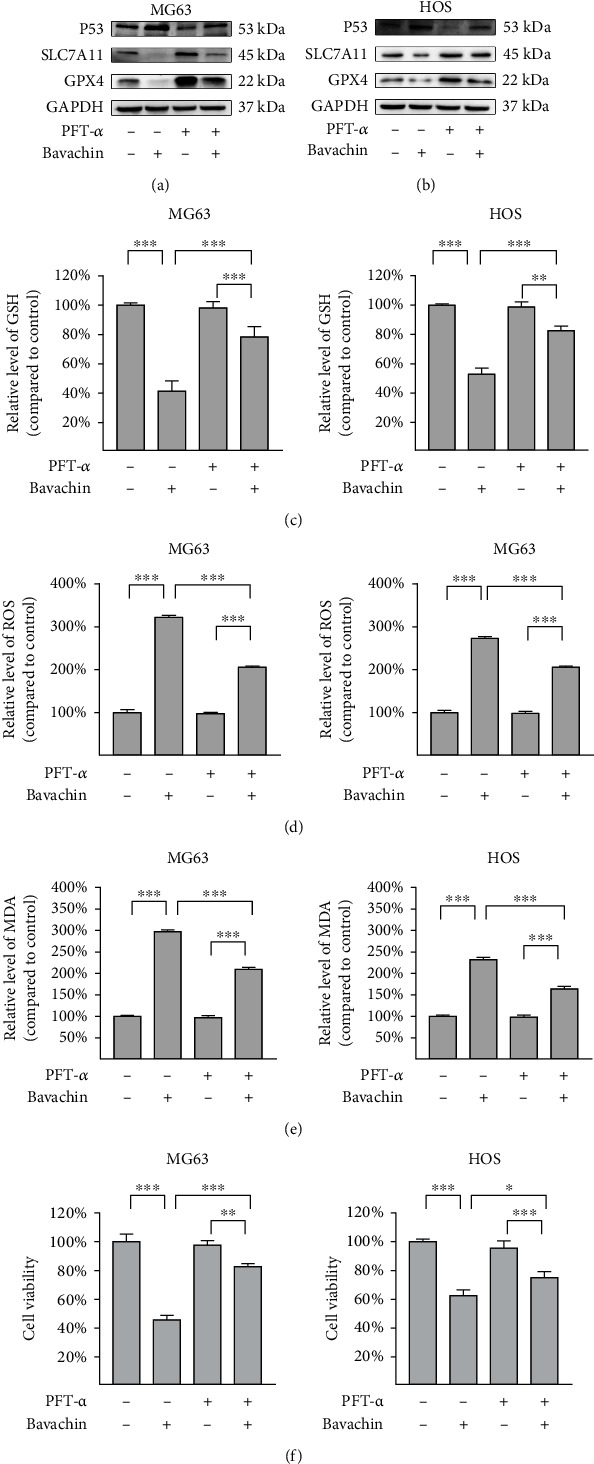
P53 inactivation upregulates SLC7A11 to alleviate bavachin-induced ferroptosis in osteosarcoma (OS) cells. OS cells were pretreated with pifithrin-*α* (PFT-*α*, 5 *μ*M) followed by treatment with bavachin (40 *μ*M, 24 h). (a, b) Western blotting shows the relative levels of P53, SLC7A11, and GPX4 protein expression. (c) PFT-*α* (5 *μ*M) restores bavachin-induced GSH depletion. (d, e) PFT-*α* reduces bavachin-induced ROS and MDA accumulation. (f) PFT-*α* recovers bavachin-induced ferroptosis in OS cells. ^∗^*p* < 0.05, ^∗∗^*p* < 0.01, and ^∗∗∗^*p* < 0.001.

Our previous experiments (Figure 4) showed that bavachin upregulated P53 and downregulated SLC7A11-induced ferroptosis in OS cells. To investigate the regulatory relationship between P53 and SLC7A11, OS cells were pretreated with 5 *μ*M PFT-*α*, a P53 inhibitor, for 0.5 h followed by treatment with 40 *μ*M of bavachin for 24 h. As shown in Figures 6(a) and 6(b), PFT-*α* inactivates P53 to upregulate the expression of SLC7A11 and GPX4 induced by bavachin. PFT-*α* also restores bavachin-induced GSH consumption (Figure 6(c)). Meanwhile, PFT-*α* significantly reduced bavachin-induced ROS and MDA accumulation (Figures 6(d) and 6(e)). Subsequently, PFT-*α* rescued bavachin-induced ferroptosis in OS cells (Figure 6(f)). In summary, these results show that P53 negatively regulates SLC7A11 and plays a promoting role in bavachin-induced ferroptosis in OS cells.

### 3.7. P-STAT3 Activation Downregulates P53 Expression to Rescue Bavachin-Induced Ferroptosis in OS Cells

**Figure 7:**
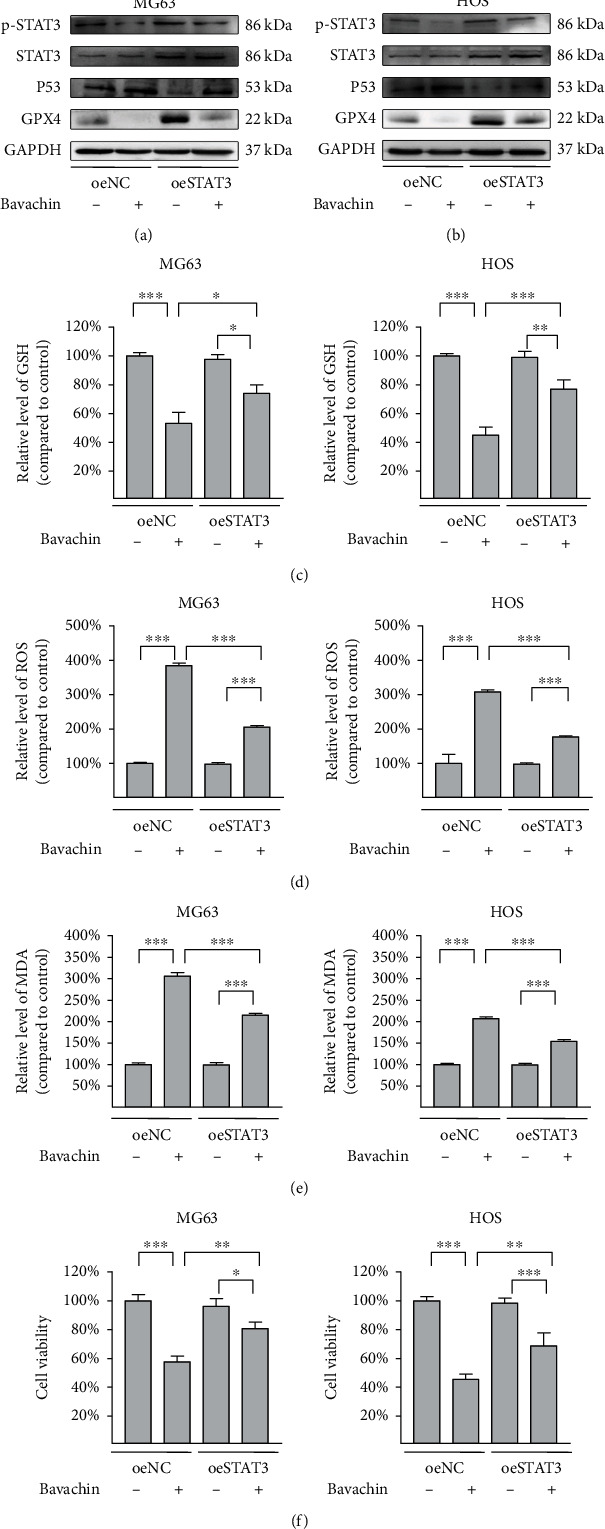
p-STAT3 activation downregulates P53 expression to rescue bavachin-induced ferroptosis in osteosarcoma (OS) cells. (a, b) Western blotting shows the relative levels of p-STAT3, STAT3, P53, and GPX4 protein expression. (c) p-STAT3 activation restores bavachin-induced GSH depletion. (d, e) p-STAT3 activation reduces bavachin-induced ROS and MDA accumulation. (f) p-STAT3 activation rescues bavachin-induced ferroptosis in OS cells. ^∗^*p* < 0.05, ^∗∗^*p* < 0.01, and ^∗∗∗^*p* < 0.001.

To evaluate whether STAT3 inhibits P53 expression to alleviate bavachin-induced ferroptosis in OS cells, OS cells were transfected with a STAT3 overexpressing plasmid, followed by treatment with 40 *μ*M bavachin for 24 h. As shown in Figures 7(a) and 7(b), compared with that in the NC group, p-STAT3 was activated, although the p-STAT3/STAT3 ratio was unchanged. p-STAT3 activation inhibited the upregulation of P53 expression induced by bavachin, while increased GPX4 expression suppressed by bavachin. p-STAT3 activation recovered GSH depletion (Figure 7(c)) and decreased the accumulation of ROS and MDA, which is stimulated by bavachin (Figures 7(d) and 7(e)). Moreover, p-STAT3 upregulation rescued bavachin-induced ferroptosis in OS cells (Figure 7(f)). As described above, p-STAT3 activation inhibits P53 expression and rescues bavachin-induced ferroptosis in OS cells.

## 4. Discussion

**Figure 8:**
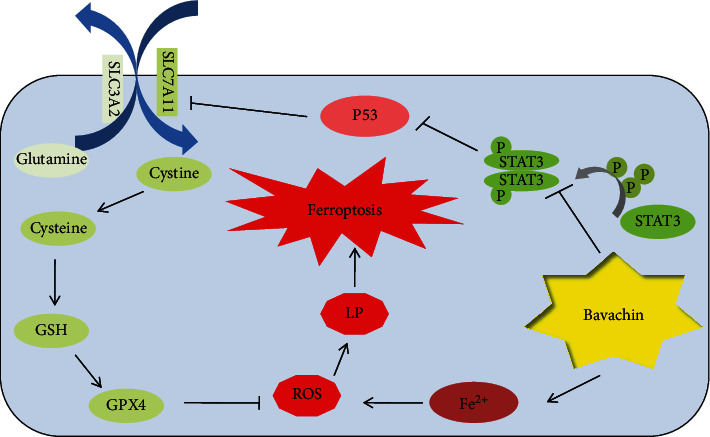
Schematic diagram displays potential mechanism of bavachin-induced ferroptosis in osteosarcoma cells. LP: lipid peroxidation.

Our study revealed that bavachin could induce OS cell death, which was reversed by iron chelator (DFO) and ferroptosis inhibitors (Fer-1, Lip-1, and Vit E). Moreover, bavachin reduced the MMP and led to mitochondrial shrinkage, increased mitochondrial membrane density, and reduced mitochondrial cristae in OS cells. Furthermore, bavachin elevated intracellular ferrous iron levels by increasing TFRC and DMT1 expression and decreasing FTH and FTL expressions. Bavachin also reduced SLC7A11 and GPX4 expression and promoted ROS and MDA accumulation by downregulating p-STAT3 to upregulate P53 expression (Figure 8).

As a new form of RCD, ferroptosis significantly differs from other types of cell death, which is due to characteristics of iron-dependence, GPX4 inhibition, and abnormal LP [5]. Moreover, ferroptosis is rescued by iron chelators and antioxidants [31, 32]. Our study consistently showed that bavachin increases ferrous iron levels and induces OS cell death, which can be rescued by Fer-1, Lip-1, Vit E, and DFO. After treatment with bavachin, morphological changes in OS cells are consistent with the classical ferroptosis morphology, which is characterised by a smaller mitochondria, higher mitochondrial membrane density, and reduced mitochondrial cristae [8]. These findings indicate that bavachin induces ferroptosis in OS cells.

Aberrant intracellular iron metabolism, especially ferrous iron overloading, is an initiating factor for ferroptosis. TFRC and DMT1 mediate ferrous iron formation and transport [33]. Previous studies suggest that downregulation of TFRC expression inhibits ferroptosis [34], and overexpression of TFRC could increase iron levels to increase ferroptosis sensitivity [35]. Similar to TFRC, DMT1 is also a positive regulator of iron accumulation, leading to ferroptosis [36]. Consistent with previous studies, our findings show that TFRC and DMT1 expressions are positively related to ferroptosis induced by bavachin. Additionally, ferritin, composed of FTL and FTH, also serves as a critical mediator of iron storage and a regulator of iron homeostasis. Previously published studies have revealed that ferritin has a two-sided effect on ferroptosis. For example, overexpression of ferritin inhibits ferroptosis in PC-12 cells via ferritinophagy [37], whereas ferroptosis occurs in glioma cells by upregulating FTL and FTH expression [38]. Although the expression of FTH and FTL in ferroptosis is diverse, our results demonstrate that bavachin downregulates FTL and FTH expressions to promote ferroptosis, which can probably be ascribed to ferritinophagy-degrading ferritin to release Fe^2+^ that is involved in ferroptosis progression [9].

Triggering LP, which is described as a process by which ROS attacks polyunsaturated fatty acids to cause dehydrogenation, is an essential event that drives ferroptosis [39]. Both ROS, generated from iron in the Fenton reaction, and MDA, the final product of LP, are contributing factors for ferroptosis activation [40]. Bavachin has been demonstrated to promote ROS generation via suppressing the MMP to trigger HepG2 cell apoptosis [28]. However, whether bavachin induces MDA has not been reported in the literature. Similarly, our results suggest that bavachin elevates the level of ROS in a dose-dependent manner and suppresses the MMP. Moreover, we found that bavachin distinctly increased the MDA content to promote ferroptosis.

The SLC7A11/GPX4 axis efficiently protects cells from ferroptoss [41]. SLC7A11, a key subunit of the system Xc-, is responsible for transporting cystine, which is reduced to cysteine for synthesis of GSH. As a vital nonenzymatic antioxidant, GSH not only scavenges free radicals but also acts as a cofactor of the GPX4-mediated reduction reaction of lipid hydroperoxides (LOOH). Inhibition of SLC7A11/GPX4, which is the most important antioxidant enzyme in the body, can trigger ferroptosis, while activation of SLC7A11/GPX4 facilitates the suppression of ferroptosis [42]. Shi et al. reported that tirapazamine induced ferroptosis in OS cells via downregulation of SLC7A11 and GPX4 expression, whereas the phenotype of cells induced by tirapazamine was reversed after overexpression of SLC7A11 [43]. Lin et al. also found that EF24, a synthetic drug, triggered ferroptosis in OS cells via inhibiting GPX4 [16]. Similarly, our results showed that bavachin led to ferroptosis in OS cells with accumulation of ROS and MDA, GSH depletion, and downregulation of GPX4 expression, while overexpression of SLC7A11 rescued OS cells from ferroptosis caused by bavachin. Therefore, we believe that bavachin triggers OS cell ferroptosis via the SLC7A11/GPX4 axis.

P53 is known as a tumour suppressor protein and can inhibit tumour cells by triggering ferroptosis [44]. Jiang et al. discovered that P53 induced ferroptosis by negatively regulating SLC7A11 to inhibit cystine transport [11]. However, a few studies have reported a contrasting view about the function of P53 in ferroptosis. In colorectal cancer cells, P53 inhibited LP and ferroptosis by binding to DPP4, while the loss of P53 led to the recovery of DPP4 to increase cell ferroptosis [45]. Tarangelo et al. found that P21, a downstream effector of P53, elevates intracellular GSH levels and GPX4 expression, leading to P53-delayed ferroptosis in some cells [46, 47]. Therefore, the two-sided functions and mechanisms of P53 in ferroptosis need to be further explored in diverse extracellular environments and cell lines. Our study showed that bavachin increased P53 expression and downregulated SLC7A11 expression to induce ferroptosis in OS cells. However, in OS cells pretreated with PFT-*α*, a P53 inhibitor, these effects were reversed. These findings suggest that bavachin triggers ferroptosis in OS cells by activating P53 to inactivate SLC7A11.

STAT3 inactivation is also associated with ferroptosis. It has been reported that STAT3 inhibits P53 to facilitate tumour progression by binding to the promoter of p53, which is an important regulator of ferroptoss [21, 48]. Qiang et al. found that increasing the expression of p-STAT3 could alleviate ferroptosis via SLC7A11 in MLE12 cells by regulating SLC7A11 [49]. In OS cells, overexpression of STAT3 aggravated ROS accumulation and ferroptosis via the Nrf2/GPX4 axis [50]. According to these studies, STAT3 appears to be a potential pharmacological target. Although bavachin was verified to inactivate p-STAT3 and increase P53 expression in multiple myeloma cells, the regulatory relationship between STAT3 and P53 has not yet been explored [30]. Our study displayed that bavachin triggers ferroptosis by inhibiting p-STAT3 but not STAT3 and increases P53 expression; however, after OS cells were transfected with the STAT3 overexpression plasmid, which simultaneously increased STAT3 and p-STAT3 expressions, bavachin no longer induced ferroptosis. It has been suggested that bavachin enables ferroptosis by inhibiting STAT3 to upregulate P53 in OS cells.

Taken together, our study displayed that bavachin triggers ferroptosis in OS cells by increasing intracellular ferrous iron levels and inhibiting the STAT3/P53/SLC7A11 axis. It suggests that bavachin could be a promising pharmaceutical for the treatment of OS.

## Data Availability

The data of this study is included within the article. The data is available from the first author (royalman1984@sina.cn) upon request.

## Supplementary Materials

Supplementary Materials Supplementary Figure 1: bavachin induces ferrous iron accumulation and ROS generation in a time-dependent manner in osteosarcoma (OS) cells.
