# Supplementary material for: Bavachin Induces Ferroptosis through the STAT3/P53/SLC7A11 Axis in Osteosarcoma Cells
Source: Oxid Med Cell Longev. 2021 Oct 18;2021:1783485. doi: 10.1155/2021/1783485 (PMC8545544; doi:10.1155/2021/1783485)
Supplement: Supplementary Materials — Supplementary Figure 1: bavachin induces ferrous iron accumulation and ROS generation in a time-dependent manner in osteosarcoma (OS) cells. [file 1783485.f1.docx]

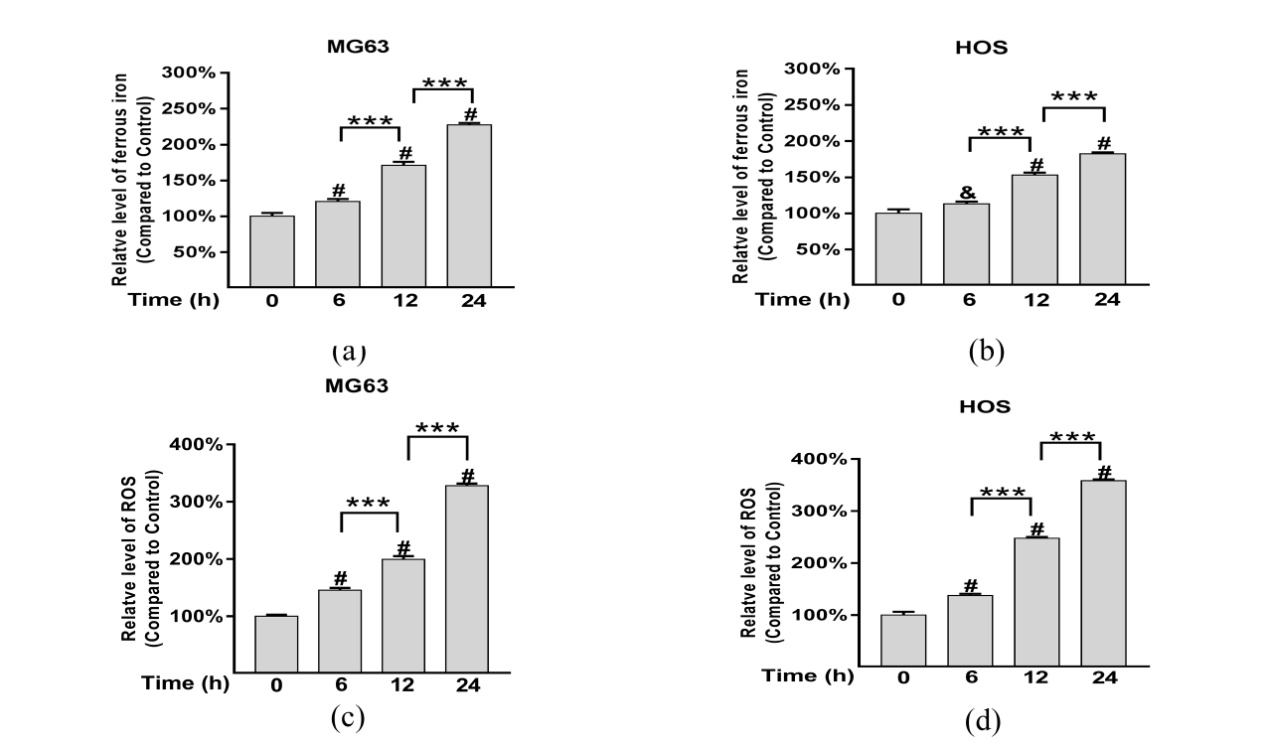


Supplementary Figure 1: Bavachin induces ferrous iron accumulation and ROS generation in a time-dependent manner in osteosarcoma (OS) cells. (a) and (b) Ferrous iron level in OS cells treated with 40 μM bavachin for 6 h, 12 h and 24 h. (c) and (d) ROS level in OS cells treated with 40 μM bavachin for 6 h, 12 h and 24 h. Data are indicated as means ± SD (n = 3). &: p < 0.01 vs. control group, #: p < 0.001 vs. control group, ***: p < 0.001.
